# Effects of Short-Term (14-Day) Intake of Sucrose and Non-Caloric Sweeteners on Glucose Regulation, Blood Lipids, Gut Hormones, Inflammation Markers, and Appetite in Healthy Adults: A Randomized Controlled Trial

**DOI:** 10.3390/nu18142337

**Published:** 2026-07-16

**Authors:** Anne Nilsson

**Affiliations:** Division Food and Pharma, Department of Process and Life Science Engineering, Lund University, P.O. Box 124, SE-221 00 Lund, Sweden; anne.nilsson@ple.lth.se

**Keywords:** non-caloric sweeteners, sucrose, steviol glycosides, saccharin, glucose regulation, insulin sensitivity, cardiometabolic risk markers, gut hormones, randomized crossover trial

## Abstract

Background/Objectives: Non-caloric sweeteners are increasingly used as alternatives to sugar to reduce energy intake, yet their metabolic effects remain controversial. This study aimed to evaluate the effects of sucrose, saccharin, and steviol glycosides on glucose regulation and cardiometabolic risk markers in healthy adults. Methods: In a randomized, double-blind, crossover trial, 39 healthy, normal-weight adults consumed beverages containing sucrose (66 g/day), saccharin (220 mg/day), or steviol glycosides (220 mg/day) for 14 days, with washout periods between interventions. Metabolic outcomes were assessed at baseline and after each intervention under fasting conditions and during a 2 h postprandial test. Primary outcomes were glucose and insulin responses; secondary outcomes included gut hormones, lipids, inflammatory markers, and subjective appetite. Results: Compared with baseline, fasting glucose increased after sucrose and saccharin, and fasting insulin increased after stevia (*p* < 0.01). All interventions increased postprandial insulin responses and reduced indices of insulin sensitivity (*p* < 0.05). Fasting PYY and GLP-2 increased following all treatments (*p* < 0.001), without differences between sweeteners. Triglycerides were higher after sucrose than saccharin (*p* < 0.05), while no differences were seen for cholesterol, apolipoproteins, or CRP. Appetite ratings were unchanged, with a trend (*p* = 0.053) towards a reduced desire to eat after stevia in the late postprandial phase. Conclusions: Short-term intake of sucrose and non-caloric sweeteners resulted in broadly similar metabolic responses, with no significant differences in glucose regulation. However, triglyceride concentrations were higher following sucrose than saccharin, whereas no consistent differences were observed across the remaining outcomes. Observed deviations from baseline should be interpreted with caution. Further long-term studies in diverse populations are warranted.

## 1. Introduction

The pandemic of lifestyle-related diseases, such as obesity and type 2 diabetes (T2D), represents a major public health and economic challenge, as these conditions are associated with an increased risk of cardiovascular diseases (CVDs) and premature mortality. Diet is one of the most important modifiable lifestyle factors in the development of cardiometabolic diseases (CMDs), making it a key target for preventive strategies.

A high intake of sucrose, particularly from sugar-sweetened beverages (SSBs), has been suggested as a contributing dietary factor to the increasing prevalence of obesity and T2D [1]. In the United States, SSB intake increased by 135% between 1977 and 2001 [2]. Although consumption has declined since the early 2000s, intake remains high across population groups [3]. SSB consumption has been consistently associated with weight gain and an increased risk of T2D in prospective cohort studies [2].

To reduce energy intake and improve metabolic health, non-caloric sweeteners (NCSs) have been widely introduced as alternatives to added sugars. These include both non-nutritive artificial sweeteners (e.g., saccharin, sucralose, and aspartame) and naturally derived sweeteners such as steviol glycosides. NCSs are now commonly incorporated into beverages and foods as sugar substitutes.

However, evidence regarding the metabolic effects of NCSs remains inconclusive. Randomized controlled trials and meta-analyses generally suggest that replacing SSBs with NCSs may reduce energy intake, body weight, and, in some cases, improve glycaemic control [4,5]. In contrast, observational studies and experimental evidence have reported associations between NCS consumption and weight gain, impaired glucose tolerance, and an increased risk of cardiometabolic diseases [6,7,8,9,10]. Consequently, the overall impact of NCSs on metabolic health remains debated.

Importantly, emerging evidence indicates that metabolic effects may differ between individual sweeteners. These differences may partly reflect differences in chemical structure and metabolism. Saccharin is a synthetic sulfamide sweetener, whereas steviol glycosides are naturally derived diterpene glycosides obtained from the Stevia rebaudiana plant.

While randomized controlled trials and meta-analyses suggest neutral or modestly beneficial effects of certain NCSs on body weight and glycaemic outcomes [4,11], experimental and animal studies indicate that specific sweeteners, such as saccharin, may adversely affect glucose metabolism under certain conditions [9,12]. In contrast, naturally derived sweeteners such as steviol glycosides have in some studies been associated with neutral or potentially beneficial effects on glucose homeostasis, although evidence from controlled human trials remains limited and inconsistent [13]. These inconsistencies may be explained by differences in chemical structure, metabolism, and interactions with physiological systems, including the gut microbiota. Thus, treating NCSs as a homogeneous group may obscure important differences between individual compounds.

Overall, current evidence does not provide consistent support for either beneficial or adverse metabolic effects of NCSs as a class [5,10,14], likely reflecting differences in study design and sweetener type. These inconsistencies highlight the need for well-controlled human intervention studies directly comparing individual NCSs under standardized conditions to clarify their metabolic effects.

Although current evidence suggests no clear effects of NCSs on circulating blood lipids in humans [15], inclusion of lipid markers enables a more comprehensive assessment of cardiometabolic risk.

Therefore, the aim of the present study was to evaluate the effects of steviol glycosides (rebaudioside A), saccharin, and sucrose on glucose regulation, inflammatory markers (C-reactive protein (CRP)), blood lipids (triglycerides, cholesterol, and apolipoproteins), gut hormones (glucagon-like peptide-1 (GLP-1), peptide YY (PYY), and glucagon-like peptide-2 (GLP-2)), and subjective appetite variables. To this end, 39 healthy middle-aged individuals with normal to slightly elevated body mass index (BMI) were included in a randomized, controlled, double-blind, crossover trial, in which each sweetener was consumed daily for 14 days and metabolic outcomes were assessed under fasting and postprandial conditions.

## 2. Materials and Methods

### 2.1. Test Participants

Recruitment began in October 2015, and all experimental procedures were completed by June 2016. The dataset has remained complete and unchanged since completion of the experimental phase.

Forty healthy volunteers (16 men and 24 women) from southern Sweden were enrolled. One male participant withdrew for personal reasons, leaving 39 participants who completed the full protocol. Baseline characteristics of the cohort are presented in Table 1.

Participants were recruited from the local community in southern Sweden through advertisements in local media, as well as through information distributed in public settings and local networks. Interested individuals contacted the research team and were subsequently screened for eligibility according to the predefined inclusion and exclusion criteria.

Healthy adults were selected to minimize the influence of pre-existing metabolic disturbances and thereby enable assessment of the metabolic responses to the sweeteners under controlled physiological conditions. Eligible participants were apparently healthy, non-smoking men and women aged 40–70 years with a normal to slightly elevated BMI (19–28 kg/m^2^).

Health status was primarily evaluated during a screening interview conducted by the investigators, together with self-reported medical history. Additional information regarding dietary habits, lifestyle factors, medication use, and diagnosed diseases was obtained using a questionnaire completed during screening. The information was used to verify eligibility according to the predefined inclusion and exclusion criteria.

All participants exhibited fasting plasma glucose values within the normal range (Table 1), confirming that the cohort was metabolically healthy.

Exclusion criteria included diagnosed hypertension, T2D, other cardiometabolic diseases, dyslipidemia, or use of medication known to affect glucose metabolism. Additional exclusion criteria were fasting plasma glucose > 6.1 mmol/L [16], food allergies, or gastrointestinal disorders.

Participants were required to consume a mixed, non-vegetarian diet in accordance with the Nordic Nutrition Recommendations and to maintain their habitual dietary patterns throughout the study. To minimize confounding dietary influences, the use of dietary supplements, including multivitamins/minerals, herbal preparations, and antioxidant-rich supplements (e.g., vitamin C, vitamin E, carotenoids, polyphenol concentrates), was prohibited for at least two weeks before the first test day and during the entire intervention period. In addition, no antibiotics or probiotics were permitted during the same time frame.

Compliance with study requirements was confirmed via self-report at screening and prior to each test day.

All participants received written and verbal information about the study procedures before enrollment, and written informed consent was obtained from each individual. The trial is reported in accordance with the CONSORT 2010 guidelines, including the extension for randomized crossover trials. A completed CONSORT checklist is provided in Supplementary File S1. The flow diagram of the study progress is available in Supplementary File S2.

### 2.2. Intervention Products

Three water-based beverages, each containing a different sweetener, were administered during the intervention. The sweeteners were:

(1) Sucrose, 66 g per day (corresponding approximately to the sugar content of two 330-mL sugar-sweetened soft drinks), (2) Steviol glycosides, 220 mg per day (>97% rebaudioside A), and (3) Saccharin, 220 mg per day.

Sucrose was included as a comparator to reflect a typical intake of sugar-sweetened beverages and to enable evaluation of the metabolic effects of replacing caloric sweeteners with NCS alternatives.

For each intervention period, the daily dose of the respective sweetener was dissolved in 1000 mL of water and prepackaged into five individual 200 mL portions prior to study initiation. Participants were instructed to consume the five portions as meal beverages throughout the day during each 14-day intervention period.

All beverages were manufactured specifically for this study by Orkla Foods Sweden (Orkla Foods Sweden, Malmö, Sweden) and were formulated to achieve broadly comparable perceived sweetness across treatments. The packaging was identical for all test beverages and labeled with codes, which were not revealed to the investigators until after completion of the analysis of the test variables.

The administered amounts of the NCSs were well below their established acceptable daily intake (ADI) values. The ADI is 4 mg/kg body weight/day for steviol glycosides and 5 mg/kg body weight/day for saccharin [17]. As all participants weighed more than 55 kg, the provided doses remained safely within these limits.

The sucrose dose was selected to represent a realistic daily intake of sugar from commonly consumed sugar-sweetened beverages.

The amounts of steviol glycosides and saccharin were selected based on commonly reported approximate sweetness equivalences relative to sucrose. The purpose was to obtain broadly comparable sweetness intensity across treatments rather than to mimic typical population intakes of individual non-caloric sweeteners. However, sweetness intensity is known to vary depending on concentration and context, and therefore exact matching of perceived sweetness between treatments cannot be guaranteed.

### 2.3. Collection and Analysis of Test Variables

Test variables in blood included markers of glucose regulation (glucose and insulin), lipid profile (triglycerides, total- LDL- and HDL cholesterol, and apolipoproteins A-1 and B), inflammatory status (CRP), and gut hormones involved in satiety and glucose regulation (total PYY and active GLP-1) and gut mucosa integrity (total GLP-2). These variables were selected because they represent different aspects of metabolic health. Glucose, insulin, HOMA-IR, and ISI_composite_ provide information on glucose regulation and insulin sensitivity. Blood lipids and apolipoproteins are established markers of cardiovascular risk, while CRP is a marker of low-grade systemic inflammation. GLP-1 is an incretin hormone involved in glucose regulation and satiety. PYY is a gut-derived hormone associated with appetite regulation and food intake. GLP-2 is involved in intestinal growth, nutrient absorption, and maintenance of gut mucosal integrity. Together, these variables provide a broad assessment of metabolic and physiological responses to the interventions.

In test situations, subjects were also asked to rank subjective satiety and hunger sensations (satiety, hunger, and desire to eat).

Fasting and postprandial measurements of physiological variables were performed at four visits: one baseline visit conducted the day before the first intervention period and three visits conducted the day after each intervention period.

Capillary blood via finger-prick was collected for determination of blood glucose (HemoCue^®^B-glucose, HemoCue AB, Ängelholm, Sweden) and serum (s-) insulin. Blood glucose and insulin were determined at fasting and every 15 min during the first hour, and thereafter every 30 min until 120 min after the start of a standardized breakfast (the breakfast is described below in the Section 2.4).

Venous blood samples were collected in the fasting state only to determine plasma (p-) concentrations of gut hormones (PYY, GLP-1 and GLP-2), CRP, and lipid profile (triglycerides, total- LDL- and HDL cholesterol, and apolipoproteins A-1 and B). Blood collecting tubes used for analysis of GLP-1, PYY and GLP-2 contained an inhibition cocktail consisting of a DPPIV-inhibitor (10 µL/mL blood) (Millipore, St. Charles, MO, USA) and aprotinin (50 µL/mL blood) (Sigma-Aldrich, St. Louis, MO, USA).

Subjective appetite sensations were registered at fasting and every half hour until 120 min, using paper sheets with a 100 mm Visual Analogue Scale (VAS).

Determination of serum insulin concentrations was performed using a solid-phase two-site enzyme immunoassay kit (Insulin ELISA 10-1113-01, Mercordia AB, Uppsala, Sweden). Plasma PYY (PYY (3–36) + PYY (1–36)) and GLP-2 (GLP-2(1–33) + GLP-2(3–33)) concentrations were determined with a competitive enzyme immunoassay (Human PYY EIA YK080 and Human GLP-2 EIA YK141, respectively; Yanaihara Institute Inc., Shizuoka, Japan). The quantitative determination of plasma GLP-1 concentrations was performed using a highly sensitive ELISA enzyme-linked immunosorbent assay kit (GLP-1 (Active 7–36) ELISA 43-GP1HU-EO1 ALPCO Diagnostics, Salem, NH, USA). Quantitative determination of plasma CRP concentrations was performed using a CRP ELISA kit (Immunodiagnostik AG, Bensheim, Germany). Routine blood tests were analyzed at the Clinical Chemistry Laboratory/Skåne University Hospital Lund, on fasting plasma (total and HDL cholesterol, triacylglycerols, apo A-1, and apo B). LDL cholesterol concentrations were calculated [18].

### 2.4. Study Design and Procedure

A randomized, double-blind crossover design was used. The treatment order was generated using a computer-based randomization sequence in Microsoft Excel.

Each test beverage was consumed daily for 14 days, whereafter blood glucose, serum insulin, and subjective appetite ratings were assessed both at fasting and during the 2 h postprandial phase following a standardized breakfast that did not contain any of the investigated sweeteners, whereas blood lipids, CRP, and gut hormones were assessed in fasting samples only.

In addition, metabolic test variables were determined at baseline the day prior to the start of the first intervention period. Thus, participants visited the clinical site in total four times for assessment of test variables.

Each participant completed three 14-day intervention periods with daily intake of the test products, one corresponding to each of the test beverages, with a minimum washout period of two weeks between interventions to minimize potential carryover effects.

On the evening before each test visit, participants consumed a standardized meal at 21:00, consisting of white wheat bread, together with 400 mL of the assigned test beverage (or water before start of the first intervention period). Additional individually standardized amounts of water, tea, or coffee, without milk or sugar, were permitted. Following the meal, participants fasted overnight until arrival at the research facility at Lund University at 07:30 the next morning.

After at least 10 min of rest, fasting measurements of all test parameters were obtained. At 08:00, a standardized breakfast providing 50 g of available starch from white wheat bread and 200 mL of water was served.

Participants were instructed to consume the breakfast at a consistent pace and to finish it within 10–12 min. The experimental procedures on each test day continued for 120 min after the start of the breakfast, with sampling conducted according to what is described above (“Collection and analysis of physiological test variables”).

To minimize potential confounding from lifestyle factors, participants were instructed to maintain their habitual diet and physical activity level throughout the study. In addition, they were asked to refrain from vigorous exercise and alcohol consumption during the 24 h preceding each test visit to minimize acute effects on the metabolic measurements.

On test mornings, participants remained seated at the research facility except when necessary.

To enhance within-subject standardization across the crossover visits, participants recorded their lunch and dinner on the day preceding the first test visit and replicated these meals before each subsequent visit. In addition, prior to and after each intervention period, participants completed structured questionnaires regarding habitual diet, intake of sweetener-containing foods, and physical activity. These records were reviewed by the investigators to verify that no major changes in dietary habits or lifestyle factors occurred during the study.

Compliance with pre-test instructions, including dietary restrictions, supplement use, physical activity, and alcohol abstention, was confirmed by self-report upon arrival on each test day.

Compliance with the intervention was monitored using daily checklists, where participants recorded the consumption of each of the five test beverage portions.

### 2.5. Calculation and Statistical Methods

Glucose tolerance and insulin responses were assessed using postprandial incremental area under the curve (iAUC) and maximum incremental peak concentrations (iPeak), calculated relative to fasting values.

iAUC was calculated for the 0–120 min postprandial period following the standardized breakfast for each subject and each test occasion, using the trapezoidal rule. iPeak was defined as the maximum postprandial increase from baseline for each subject and test occasion.

The composite insulin sensitivity index (ISI_composite_) was used to determine insulin sensitivity. However, the index was modified since the breakfast (WWB) included 50 g available carbohydrates instead of 75 g of glucose as in the original formula. ISI_composite_ was calculated using the formula: 10,000/square root of [f-glucose (mg/dL) * f-insulin (µU/mL) * mean glucose concentrations 0–120 min (mg * min/dL) * mean insulin concentrations 0–120 min (µU * min/mL)] [19]. The mean glucose- and insulin concentrations included in the formula were based on blood glucose and s-insulin values measured every 30 min in the postprandial phase after the standardized breakfast.

Homeostatic model assessment for insulin resistance (HOMA-IR) was calculated using the formula [f-glucose (mmol/L) * f-insulin (mU/L)/22.5] [20].

Graphical representation of incremental curves and calculation of areas were performed in GraphPad Prism (version 6; GraphPad Software, San Diego, CA, USA). To provide a comprehensive overview, curves showing absolute (actual) blood glucose and serum insulin concentrations are also presented. Differences between treatments were assessed using analysis of variance (ANOVA; general linear model, MINITAB, release 17; Minitab Inc., State College, PA, USA), in which subject was included as a fixed factor to account for the crossover design, with each participant serving as his/her own control. Post hoc comparisons were performed using Tukey’s post hoc test for pairwise comparisons in MINITAB (release 17; Minitab Inc., State College, PA, USA), which accounts for multiple comparisons within each outcome variable.

The suitability of the statistical models was assessed before the analyses were performed. Normality of residuals was assessed using normal probability plots and histograms; residuals versus fitted values and residuals versus observation order were examined to assess homoscedasticity and independence. When indicated by visual inspection, the Anderson–Darling test was applied. For test variables where model assumptions were not met, a Box–Cox transformation was performed on the data prior to ANOVA analysis.

Thus, the model represents a randomized block design and accounts for within-subject variability. Carryover effects were considered unlikely due to the washout periods between interventions.

Secondary outcomes were analyzed in an exploratory manner and were not adjusted for multiple testing.

The primary outcome was the change in blood glucose concentrations (iAUC 0–120 min) after the standardized breakfast, reflecting effects on glucose tolerance. Power calculation was performed in MINITAB. Assuming a mean difference of 42 mmol * min/L between test products and baseline, a standard deviation of 67 mmol * min/L [21], α = 0.05, and power (1 − β) = 0.8, 22 participants were required. Among additional measures, changes in LDL-cholesterol were also considered. Assuming a 0.5 mmol/L (approximately 10%) difference between treatments and a 0.97 SD [22], with α = 0.05 and 1 − β = 0.8, a minimum of 30 participants was required. Therefore, although glucose iAUC was defined as the primary outcome, the final sample size was based on the larger sample size requirement for LDL-cholesterol. To account for potential dropouts, the sample size was increased to 40 participants. Apart from the primary outcome, all other analyses should be considered exploratory and interpreted accordingly. Baseline characteristics are presented as means ± SD, while all other results are expressed as means ± SEM. Statistical significance was set at *p* < 0.05.

## 3. Results

### 3.1. Glucose Regulation

**Figure 1:**
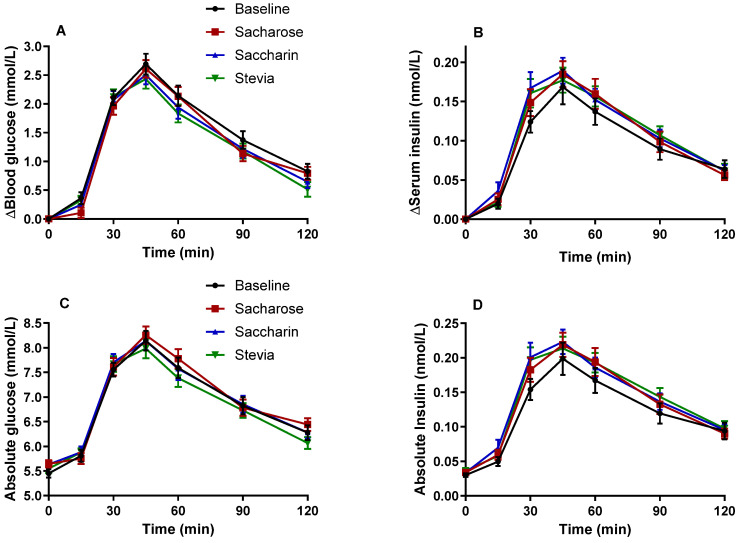
(**A**–**D**). Mean incremental changes (Δ) in blood glucose (**A**) and insulin (**B**) concentrations following a standardized breakfast at baseline and after 14 days of interventions with stevia, saccharin and sucrose, respectively. (**C**) (glucose) and (**D**) (insulin) display results in absolute concentrations.

No significant differences were observed between the sweeteners (saccharose, saccharin, stevia) in any of the glucose regulation markers (fasting and postprandial blood glucose and serum insulin, HOMA-IR, ISI_coposite_ *p* > 0.05) (Table 2, Figure 1A).

However, differences from pre-study baseline values were observed for some of the test markers. These comparisons should be interpreted with caution, as baseline was not part of the randomized crossover design.

Small but significant increases in fasting glucose from baseline were observed after 14 days of intervention with sucrose (3.7%, *p* < 0.01) and saccharin (3.3%, *p* < 0.05).

Additionally, in comparison with baseline values, fasting insulin concentrations were significantly increased (20%) after stevia intake compared with baseline (*p* < 0.05; Table 2).

Postprandial insulin responses (insulin iAUC 0–120 min) were also significantly increased compared with baseline for sucrose (*p* < 0.05), saccharin (*p* < 0.01), and stevia (*p* < 0.01) (Figure 1B).

Similarly, insulin iPeak was significantly increased following interventions (*p* < 0.01), with higher values observed for saccharin and stevia (*p* < 0.01), and sucrose (*p* < 0.05), compared with baseline.

Absolute postprandial glucose and insulin profiles are presented in Figure 1C,D.

Furthermore, insulin resistance, as assessed by HOMA-IR, was higher after stevia intake compared with baseline (+21%, *p* < 0.05). Insulin sensitivity, assessed by ISI_composite_, also differed relative to baseline, with reductions from baseline observed after saccharin (−25%, *p* < 0.05) and stevia (−27%, *p* < 0.01), while a non-significant trend was observed after sucrose (*p* = 0.074).

### 3.2. Intestinal Hormones, Blood Lipids, and Inflammatory Markers

No significant differences in GLP-1, PYY, or GLP-2 were observed between the sweeteners (*p* > 0.05; Table 3). Compared with baseline, all sweeteners significantly increased fasting concentrations of PYY (sucrose + 19%, saccharin + 24%, stevia + 15%; *p* < 0.001) and GLP-2 (sucrose + 11%, saccharin + 17%, stevia + 11%; *p* < 0.001). Triglyceride concentrations were higher after sucrose intake compared with saccharin (+15%, *p* < 0.05; Table 3). However, no significant differences in triglycerides were observed compared with baseline after any of the interventions.

No significant effects were detected on total, LDL, or HDL cholesterol; apolipoproteins (ApoA-1 and ApoB); or C-reactive protein (CRP) (*p* > 0.05) (Table 3).

### 3.3. Subjective Appetite Ratings and Weight

The data regarding subjective appetite ratings are displayed in Table 4. No significant differences were observed compared with baseline or between treatments in any of the subjective appetite variables (satiety, hunger, and desire to eat) at fasting or in mean sensations during the 0–120 min postprandial period.

At the later postprandial period (120 min) a trend towards a treatment effect was observed for desire to eat (ANOVA, *p* = 0.053), with lower ratings after stevia intake compared with baseline (−16%; Tukey’s test *p* = 0.07).

Overall, no significant differences were observed between the sweeteners in any of the subjective appetite variables (*p* > 0.05).

## 4. Discussion

The present randomized, double-blind crossover study investigated the metabolic effects of 14-day intake of sucrose, saccharin, and steviol glycosides in healthy, normal-weight to slightly overweight adults. The main finding was that no significant differences were observed between the sweeteners in glucose regulatory variables, and only minor and isolated differences were detected across the broader range of metabolic outcomes.

When compared with baseline values obtained prior to the first intervention period, several metabolic variables were altered following all three treatments. These included increases in fasting glucose (sucrose and saccharin), fasting insulin (stevia), postprandial insulin responses, and indices of insulin resistance, together suggesting modest reductions in insulin sensitivity. However, these baseline comparisons should be interpreted with caution, as baseline was not included in the randomized crossover structure.

The absence of differences between sucrose and non-caloric sweeteners in glucose regulation suggests that, under the present experimental conditions, replacement of sucrose with non-caloric sweeteners does not confer measurable short-term metabolic benefits or adverse effects in healthy individuals. This finding is in line with previous randomized and controlled studies, as well as systematic reviews, reporting largely neutral effects of non-nutritive sweeteners on glycaemic control and lipid metabolism [5,10,13,14,15].

Despite the overall similarity between treatments, some isolated differences were observed. Triglyceride concentrations were higher following sucrose intake compared with saccharin, although no differences were observed relative to baseline. This finding may reflect differences in the metabolic handling of caloric versus non-caloric sweeteners, but the absence of consistent effects across lipid markers suggests limited clinical relevance in the present short-term setting.

In addition, a trend towards reduced desire to eat in the late postprandial phase was observed after stevia intake compared with baseline. Appetite regulation at later time points may influence subsequent energy intake, and this observation could therefore be of potential physiological interest. However, given the borderline statistical significance and lack of effects on other appetite measures, this finding should be interpreted cautiously. Moreover, given the exploratory nature of the secondary outcomes and the number of statistical tests performed, this observation requires confirmation in future studies. Fasting concentrations of PYY and GLP-2 increased following all interventions, without differences between sweeteners. These responses may reflect adaptive changes in gut hormone secretion in response to repeated exposure to sweet-tasting beverages. The physiological implications of these alterations remain unclear but may involve anticipatory or regulatory mechanisms related to energy intake and gastrointestinal function, potentially reflecting responses to repeated exposure to sweet taste with and without caloric content.

A major strength of the study is the randomized, double-blind crossover design, which minimizes inter-individual variability and allows robust within-subject comparisons between treatments. The use of beverages matched for perceived sweetness reduces sensory confounding, and the comprehensive assessment of metabolic outcomes, including glucose regulation, gut hormones, lipids, and subjective appetite, provides a broad characterization of cardiometabolic responses.

Several limitations should be considered. The present study should be regarded as a controlled physiological investigation in metabolically healthy adults, and the findings should therefore not be extrapolated to populations with cardiometabolic disease or increased cardiometabolic risk.

First, baseline measurements were obtained prior to the start of the intervention and were not included in the randomization scheme. Consequently, comparisons between baseline and intervention periods should be interpreted with caution, as they may be influenced by time-dependent factors unrelated to the treatments. Importantly, however, the randomized crossover design ensures that comparisons between the sweeteners are internally valid.

Second, the intervention duration of 14 days may be insufficient to detect longer-term effects on insulin sensitivity, body weight regulation, or lipid metabolism. Third, the study population consisted of healthy, normal-weight to slightly overweight individuals, which may limit the generalizability of the findings to populations with impaired glucose metabolism or increased cardiometabolic risk. Furthermore, volunteer bias cannot be excluded, since participation was voluntary and individuals enrolling in nutrition intervention studies may be more health-conscious than the general population.

In addition, dietary intake outside the intervention beverages was not strictly controlled, and the study may have been underpowered to detect small differences in some secondary outcomes. While dietary intake during the full intervention periods was not strictly controlled, efforts were made to verify consistency in dietary habits and physical activity across study periods, and to standardize conditions prior to each test visit, which is particularly relevant for minimizing short-term effects related to prior meals. However, as dietary intake and lifestyle factors were not strictly controlled throughout the intervention periods and relied on self-report, residual confounding cannot be excluded. In addition, compliance with the intervention was assessed using self-reported daily checklists rather than objective measures such as returned container counts. These limitations are particularly relevant for outcomes with small effect sizes.

Finally, although the beverages were designed to have broadly comparable perceived sweetness, no formal sensory validation was performed, and differences in perceived sweetness, palatability, mouthfeel, and aftertaste between treatments cannot be excluded.

## 5. Conclusions

In conclusion, 14-day intake of sucrose, saccharin, and steviol glycosides resulted in largely similar metabolic responses in healthy adults, with no significant differences in glucose-regulatory outcomes. However, a modest increase in triglyceride concentrations was observed with sucrose compared with saccharin.

Changes observed relative to baseline should be interpreted with caution due to limitations in the study design.

Future studies should investigate the long-term metabolic effects of NCSs in diverse population groups, including individuals with insulin resistance or T2D, and further explore the mechanisms by which repeated exposure to sweet taste may influence metabolic regulation, preferably in well-controlled studies of longer duration.

## Figures and Tables

**Table 1:** Characteristics of the participants who completed the study ^1^.

	Total Cohort	Male	Female
*n*	39	15	24
Age (years)	59.3 ± 10.1	58.5 ± 10.3	59.9 ± 10.1
BMI (kg/m^2^)	24.4 ± 2.60	25.5 ± 3.10	23.80 ± 2.04
Systolic BT (mm Hg)	127 ± 18	132 ± 17	125 ± 17
Diastolic BT (mm Hg)	86 ± 12	87 ± 12	85 ± 12
Fasting p-glucose (mmol/L)	5.45 ± 0.52	5.52 ± 0.49	5.40 ± 0.54
Fasting s-insulin (nmol/L)	0.030 ± 0.016	0.033 ± 0.018	0.028 ± 0.015
HOMA-IR ^2^	1.06 ± 0.61	1.22 ± 0.63	0.97 ± 0.57

^1^ All values are mean ± SD, ^2^ HOMA-IR: homeostatic model of insulin resistance.

**Table 2:** Glucose homeostasis variables at baseline and after 14-day interventions with different sweeteners ^1^.

Test Variables	Baseline	Sucrose	Saccharin	Stevia
Fasting glucose (mmol/L)	5.45 ± 0.09 ^a^	5.65 ± 0.07 ^b^	5.63 ± 0.07 ^b^	5.55 ± 0.09 ^ab^
Glucose iAUC (mmol * time/L)	181.3 ± 12.4 ^a^	166.7 ± 10.8 ^a^	164.9 ± 11.8 ^a^	159.2 ± 10.1 ^a^
Glucose iPeak (mmol/L)	2.97 ± 0.16 ^a^	2.86 ± 0.15 ^a^	2.86 ± 0.15 ^a^	2.75 ± 0.14 ^a^
Fasting insulin (nmol/L)	0.030 ± 0.003 ^a^	0.034 ± 0.003 ^ab^	0.034 ± 0.002 ^ab^	0.036 ± 0.003 ^b^
Insulin iAUC (nmol * time/L)	11.43 ± 1.28 ^a^	13.01 ± 1.11 ^b^	13.34 ± 1.09 ^b^	13.05 ± 0.88 ^b^
Insulin iPeak (nmol/L)	0.224 ± 0.023 ^a^	0.258 ± 0.022 ^b^	0.268 ± 0.020 ^b^	0.265 ± 0.019 ^b^
HOMA-IR	1.07 ± 0.10 ^a^	1.23 ± 0.11 ^b^	1.21 ± 0.074 ^b^	1.31 ± 0.096 ^b^
ISI_composite_	15.37 ± 1.93 ^a^	11.48 ± 0.94 ^b^	11.12 ± 1.16 ^b^	11.16 ± 1.15 ^b^

^1^ All values are mean ± SEM. *n* = 37 for analyses of ISI_composite_, *n* = 38 for the remaining test variables. Values in the same row with different superscript letters are significantly different, *p* < 0.05 (ANOVA, followed by Tukey’s test). iAUC, incremental area under the curve; iPeak, incremental peak; HOMA-IR, homeostatic model of insulin resistance; ISI_composite_, insulin sensitivity index.

**Table 3:** Intestinal hormones, blood lipids, and CRP concentrations at baseline and after fourteen days intervention with different sweeteners.

Test Variable	Baseline	Sucrose	Saccharin	Stevia
f-GLP-1 (pmol/L) (*n* = 36)	3.26 ± 0.55 ^a^	2.71 ± 0.45 ^a^	2.83 ± 0.47 ^a^	3.0 ± 0.61 ^a^
f-PYY (ng/L) (*n* = 38)	357 ± 42 ^a^	425 ± 44 ^b^	444 ± 57 ^b^	412.0 ± 43 ^b^
f-GLP-2 (ng/mL) (*n* = 36)	4.68 ± 0.14 ^a^	5.28 ± 0.16 ^b^	5.55 ± 0.18 ^b^	5.50 ± 0.18 ^b^
f-Triglycerides (mmol/L)	1.09 ± 0.07 ^ab^	1.16 ± 0.10 ^a^	1.01 ± 0.06 ^b^	1.06 ± 0.08 ^ab^
f-Cholesterol-total (mmol/L)	5.69 ± 0.16 ^a^	5.58 ± 0.16 ^a^	5.5 ± 0.17 ^a^	5.59 ± 0.18 ^a^
f-LDL-cholesterol (mmol/L)	3.38 ± 0.16 ^a^	3.39 ± 0.15 ^a^	3.39 ± 0.16 ^a^	3.39 ± 0.16 ^a^
f-HDL-cholesterol (mmol/L)	1.69 ± 0.08 ^a^	1.67 ± 0.09 ^a^	1.69 ± 0.09 ^a^	1.69 ± 0.09 ^a^
f-ApoA-1 (g/L)	1.76 ± 0.05 ^a^	1.71 ± 0.05 ^a^	1.71 ± 0.05 ^a^	1.71 ± 0.05 ^a^
f-ApoB (g/L)	1.00 ± 0.04 ^a^	1.00 ± 0.04 ^a^	0.98 ± 0.04 ^a^	0.99 ± 0.05 ^a^

All values are mean ± SEM. *n* = 39 unless otherwise indicated in the table. f; fasting. Values in the same row with different superscript letters are significantly different, *p* < 0.05 (ANOVA, followed by Tukey’s test).

**Table 4:** Subjective appetite sensations and weight at baseline and after fourteen days intervention with different sweeteners.

Test Variables	Baseline	Sucrose	Saccharin	Stevia
Satiety, 0–120 min (cm)	4.3 ± 0.3	4.4 ± 0.2	4.6 ± 0.2	4.6 ± 0.2
Hunger, 0–120 min (cm)	4.5 ± 0.4	4.3 ± 0.3	4.3 ± 0.3	4.3 ± 0.3
Desire to eat, 0–120 min (cm)	5.3 ± 0.4	4.6 ± 0.3	4.8 ± 0.3	4.8 ± 0.3
Satiety at 120 min (cm)	3.8 ± 0.4	4.3 ± 0.4	4.2 ± 0.3	4.3 ± 0.3
Hunger at 120 min (cm)	5.3 ± 0.5	4.8 ± 0.4	4.7 ± 0.3	4.9 ± 0.3
Desire to eat at 120 min (cm)	6.0 ± 0.5 ^#^	5.1 ± 0.4	5.1 ± 0.4	5.0 ± 0.4 ^#^
Weight (kg)	73.5 ± 2.0	73.7 ± 2.1	73.6 ± 2.2	73.8 ± 2.0

All values are mean ± SEM. *n* = 39. No significant differences were observed. #; *p* = 0.07 (Tukey’s test).

## Data Availability

The datasets analyzed during this study are available from the corresponding author on reasonable request.

## Supplementary Materials

The following supporting information can be downloaded at: https://www.mdpi.com/article/10.3390/nu18142337/s1, Supplementary File S1: CONSORT 2010 checklist; Supplementary File S2: Flow diagram.
