# Supplementary material for: Effects of Short-Term (14-Day) Intake of Sucrose and Non-Caloric Sweeteners on Glucose Regulation, Blood Lipids, Gut Hormones, Inflammation Markers, and Appetite in Healthy Adults: A Randomized Controlled Trial
Source: Nutrients. 2026 Jul 16;18(14):2337. doi: 10.3390/nu18142337 (PMC13415396; doi:10.3390/nu18142337)
Supplement: Supplementary file 1 [file nutrients-18-02337-s001.zip › nutrients-4400389-supplementary file S1.pdf]

Supplementary file S1

CONSORT 2010 checklist of information to include when reporting a randomised trial*

Section/Topic	Item No	Checklist item	Reported on page No
**Title and abstract**			
	1a	Identification as a randomised trial in the title	Yes
	1b	Structured summary of trial design, methods, results, and conclusions (for specific guidance see CONSORT for abstracts)	Yes
**Introduction**			
Background and objectives	2a	Scientific background and explanation of rationale	2-3
	2b	Specific objectives or hypotheses	3
**Methods**			
Trial design	3a	Description of trial design (such as parallel, factorial) including allocation ratio	5 (2.4. Study design and Procedure)
	3b	Important changes to methods after trial commencement (such as eligibility criteria), with reasons	Not applicable
Participants	4a	Eligibility criteria for participants	3 (2.1. Test participants)
	4b	Settings and locations where the data were collected	5 (2.4. Study design and Procedure)
Interventions	5	The interventions for each group with sufficient details to allow replication, including how and when they were actually administered	4 (2.2. Intervention products)
Outcomes	6a	Completely defined pre-specified primary and secondary outcome measures, including how and when they were assessed	4,5 (2.3. Collection and analysis of test variables and 2.5. Calculation and statistical

			methods)
	6b	Any changes to trial outcomes after the trial commenced, with reasons	Not applicable
Sample size	7a	How sample size was determined	6 (2.5. Calculation and statistical methods)
	7b	When applicable, explanation of any interim analyses and stopping guidelines	Not applicable
Randomisation:			5 (Calculation and statistical methods)
Sequence generation	8a	Method used to generate the random allocation sequence	
	8b	Type of randomisation; details of any restriction (such as blocking and block size)	5 (2.4. Study design and Procedure)
Allocation concealment mechanism	9	Mechanism used to implement the random allocation sequence (such as sequentially numbered containers), describing any steps taken to conceal the sequence until interventions were assigned	4 (Intervention products)
Implementation	10	Who generated the random allocation sequence, who enrolled participants, and who assigned participants to interventions	**Sequence:** blinded study investigator. **Enrollment:** blinded study investigator. **Assignment:** blinded lab assistant using pre-packaged , code-labeled, visually identical packagings.

Blinding	11a	If done, who was blinded after assignment to interventions (for example, participants, care providers, those assessing outcomes) and how	See q 10
	11b	If relevant, description of the similarity of interventions	4 (2.2. Intervention products. Identical packages and products matched for sweetenes)
Statistical methods	12a	Statistical methods used to compare groups for primary and secondary outcomes	6 (Calculation and statistical methods)
	12b	Methods for additional analyses, such as subgroup analyses and adjusted analyses	Not applicable
**Results**			
Participant flow (a diagram is strongly recommended)	13a	For each group, the numbers of participants who were randomly assigned, received intended treatment, and were analysed for the primary outcome	3 (2.1. Test participants) 40 were randomised, 39 were analysed. See also Flow diagram in supplementar y file 2
	13b	For each group, losses and exclusions after randomisation, together with reasons	One, see Flow diagram, losses due to personal reasons
Recruitment	14a	3	3 (2.1. Test participants.

	14b	Why the trial ended or was stopped	3. completed as planned
Baseline data	15	A table showing baseline demographic and clinical characteristics for each group	Table 1, P 3-4
Numbers analysed	16	For each group, number of participants (denominator) included in each analysis and whether the analysis was by original assigned groups	39, crossover, all participants
Outcomes and estimation	17a	For each primary and secondary outcome, results for each group, and the estimated effect size and its precision (such as 95% confidence interval)	6-9 (3-Results)., Tables 2–4; Figures 1A–D
	17b	For binary outcomes, presentation of both absolute and relative effect sizes is recommended	Not applicable
Ancillary analyses	18	Results of any other analyses performed, including subgroup analyses and adjusted analyses, distinguishing pre-specified from exploratory	Not applicable
Harms	19	All important harms or unintended effects in each group (for specific guidance see CONSORT for harms)	No adverse events
**Discussion**			
Limitations	20	Trial limitations, addressing sources of potential bias, imprecision, and, if relevant, multiplicity of analyses	10
Generalisability	21	Generalisability (external validity, applicability) of the trial findings	9-10 Discussion, Conclusions
Interpretation	22	Interpretation consistent with results, balancing benefits and harms, and considering other relevant evidence	9-10 Discussion, Conclusions
**Other information**			
Registration	23	Registration number and name of trial registry	P. 10. ClinicalTrials. gov (Identifier: (NCT02580110).
Protocol	24	Where the full trial protocol can be accessed, if available	P. 10. The full trial protocol

			is available from the corresponding author upon reasonable request
Funding	25	Sources of funding and other support (such as supply of drugs), role of funders	P. 10. Antidiabetic Food Centre, a VINNOVA VINN Excellence Center at Lund University, funded the execution of the study

*We strongly recommend reading this statement in conjunction with the CONSORT 2010 Explanation and Elaboration for important clarifications on all the items. If relevant, we also recommend reading CONSORT extensions for cluster randomised trials, non-inferiority and equivalence trials, non-pharmacological treatments, herbal interventions, and pragmatic trials. Additional extensions are forthcoming: for those and for up to date references relevant to this checklist, see www.consort-statement.org.
