# Supplementary figures and images for: Effects of Short-Term (14-Day) Intake of Sucrose and Non-Caloric Sweeteners on Glucose Regulation, Blood Lipids, Gut Hormones, Inflammation Markers, and Appetite in Healthy Adults: A Randomized Controlled Trial
Source: Nutrients. 2026 Jul 16;18(14):2337. doi: 10.3390/nu18142337 (PMC13415396; doi:10.3390/nu18142337)

Supplementary file S2, Flow diagram

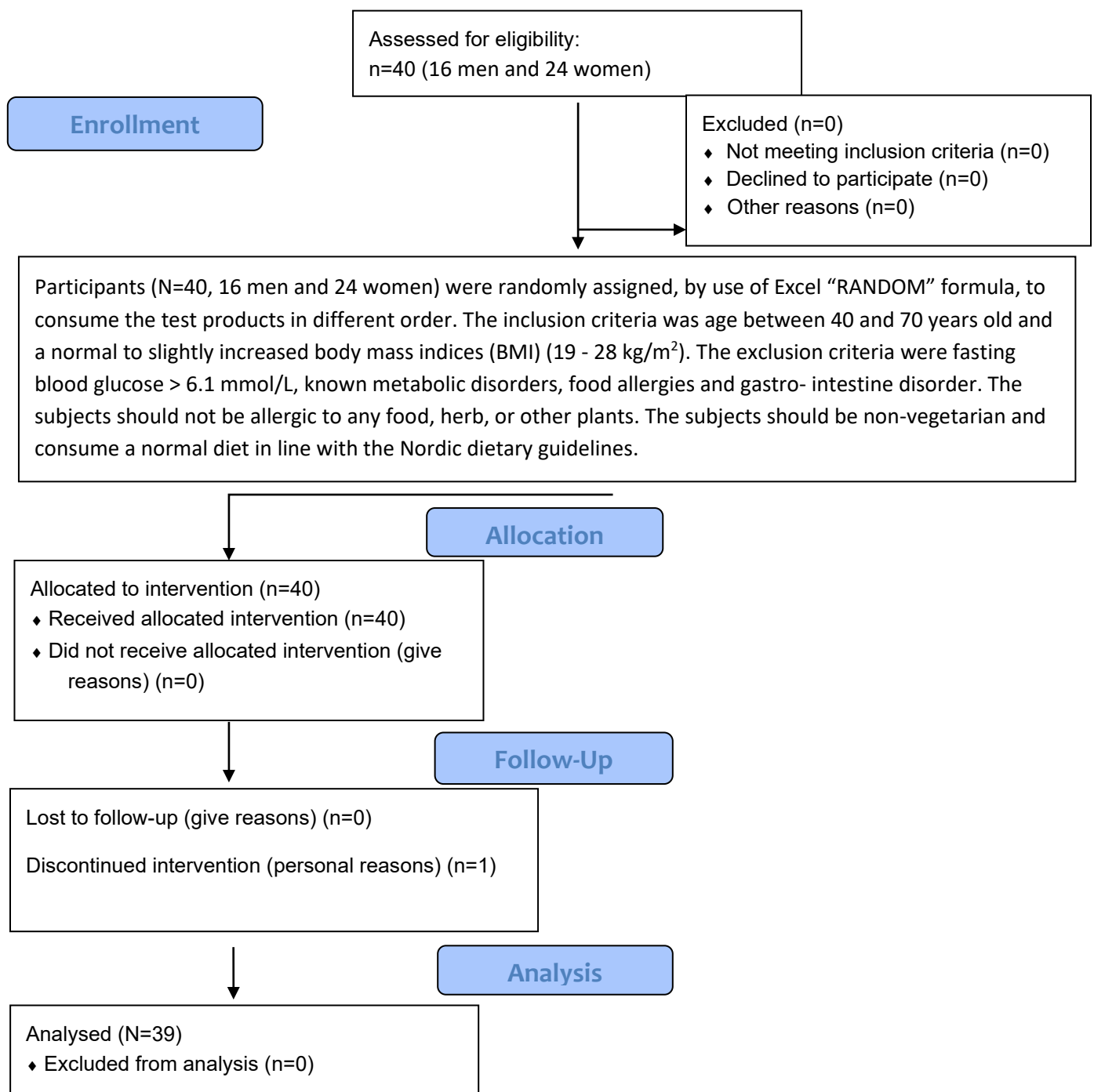

Supplement: Supplementary file 1 [file nutrients-18-02337-s001.zip › nutrients-4400389-supplementary file S2.pdf]
